# Racial Composition of Past and Current Social Environments and Health Literacy

**DOI:** 10.3928/24748307-20240719-01

**Published:** 2024-07

**Authors:** Jemar R. Bather, Feng Liu, Melody S. Goodman, Kimberly A. Kaphingst

## Abstract

**Background:**

Research is needed to understand the impact of social determinants of health on health literacy throughout the life course. This study examined how racial composition of multiple past and current social environments was related to adults' health literacy.

**Methods:**

In this study, 546 adult patients at a primary care clinic in St. Louis, Missouri, completed a self-administered written questionnaire that assessed demographic characteristics and a verbally administered component that assessed health literacy with the Rapid Estimate of Adult Literacy in Medicine – Revised (REALM-R) and Newest Vital Sign (NVS), and self-reported racial composition of six past and four current social environments. Multilevel logistic regression models were built to examine the relationships between racial composition of past and current social environments and health literacy.

**Results:**

Most participants identified as Black or multiracial (61%), had a high school diploma or less (54%), and household income <$20,000 (72%). About 56% had adequate health literacy based on REALM-R and 38% based on NVS. In regression models, participants with multiple past white environments (e.g., locations/conditions in which most of the people who live, go to school, work, and have leisure time are White) and (vs. 0 or 1) were more likely to have adequate health literacy based on REALM-R (adjusted odds ratio [aOR] = 1.79; 95% confidence interval [CI]: 1.04–3.07). Similarly, participants who had multiple past white social environments were more likely (aO*R* = 1.94, 95% CI: 1.15–3.27) to have adequate health literacy based on NVS than those who had not. The racial composition of current social environments was not significantly associated with health literacy in either model.

**Conclusions:**

Racial composition of past, but not current, educational and residential social environments was significantly associated with adult health literacy. The results highlight the importance of examining the impact of social determinants over the life course on health literacy. The findings suggest that policies ensuring equitable access to educational resources in school and community contexts is critical to improving equitable health literacy. [***HLRP: Health Literacy Research and Practice*. 2024;8(3):e130–e139.**]

## Abstract (plain language summary)

**Plain Language Summary: **We studied how the racial make-up of past and current places where people live, work, and go to school were related to their health literacy as adults. We found that the racial make-up of past places, but not current places, was related to health literacy. Our results show the need to study the impact of childhood places on health literacy.

Health literacy has been shown to have a critical impact on health outcomes and health disparities across a range of health conditions (Berkman et al., 2011). In studies examining the effects of health literacy, the construct has been traditionally operationalized as an individual-level variable related to how patients operate within the health care system, particularly in the United States (Malloy-Weir et al., 2016). However, public health models have expanded our conceptualization of health literacy to encompass dimensions that go beyond individual-level competencies and the medical context (Koh & Rudd, 2015; Sørensen et al., 2012). Sørensen et al. (2012) proposed a multilevel model of health literacy that integrates the individual-level competencies of accessing, understanding, appraising, and applying health information with contextual demands of the health care setting, disease prevention systems, and health promotion in the community (Sørensen et al., 2012).

Together with the recognition of the importance of system- and community-level contexts, there has been increasing interest in the relationship between health literacy and social determinants of health (Nutbeam & Lloyd, 2021; Schillinger, 2021). Early frameworks of health literacy highlighted that individual-level characteristics such as race, ethnicity, education, and age were upstream of health literacy (Paasche-Orlow & Wolf, 2007), and many prior research studies showed that health literacy was significantly correlated with these demographic factors (Kutner et al., 2006; Nutbeam & Lloyd, 2021; Paasche-Orlow et al., 2005). However, mechanisms underlying these correlations have received less research (Khoong & Schillinger, 2020). One analysis of nationally representative survey data examined the upstream social determinants of health literacy, finding correlations between health literacy and objective social status indicators, relational social status indicators, and social resources (Rikard et al., 2016). Other prior research has examined whether health literacy might mediate the impact of upstream social determinants of health on disparities in health outcomes (Gwynn et al., 2016; Seibert et al., 2019). For example, in a longitudinal study of adults with persistent asthma, Seibert et al. (2019) found that racial disparities in asthma-related emergency department visits were partially mediated through health literacy (Seibert et al., 2019). A 2015 systematic review found evidence for a mediating function of health literacy in the pathway between racial and ethnic disparities and self-rated health status, and a potential mediation effect of health literacy between racial and ethnic disparities and medication adherence (Mantwill et al., 2015). Schillinger (2020) proposed that health literacy might partially mediate the impact of social determinants of health on health outcomes and health disparities, with health literacy also impacted by structural resources and organizational health literacy (Schillinger, 2020). Reviews of this area have highlighted the need to examine how system-level factors affect health literacy to better understand its relationship with social determinants of health (Nutbeam & Lloyd, 2021; Schillinger, 2020).

One area in which additional research is needed is understanding the impact of various social determinants of health at different timepoints. Prior research on the relationships between social determinants of health and health literacy has generally focused on current contexts. However, our previous research demonstrated the importance of considering the effects of both past and current contexts on health literacy. In a prior study, we found that the racial composition of an individual's junior high school and the racial composition of their current neighborhood were both associated with adult health literacy (Goodman et al., 2012). While educational attainment has consistently been shown to be associated with health literacy in adults (Kutner et al., 2006; Paasche-Orlow et al., 2005), other studies are needed to investigate how both educational and neighborhood resources in childhood might impact adult health literacy, and whether these effects are independent of the impact of structural resources experienced in adulthood.

The purpose of this study was therefore to examine how racial composition of multiple past and current environments was related to adults' health literacy in a highly residentially segregated urban area in the U.S. Conceptual work by Jones (2000) highlights that racism structures the social organization of people, power and resources in the United States and is codified in structural custom, practice, and law (Jones, 2000). Because racial residential segregation results in the differential allocation of socioeconomic and other health-related resources, determines access to health-promoting resources and services, and constrains individual health choices that affect health risks, it has been described as underlying Black-White disparities in health status (LaVeist, 2003; Logan et al., 2004; Massey & Denton, 1993; Williams & Collins, 2001). However, the impact of racial residential segregation on health literacy has received limited attention. Based on prior work, we hypothesized that individuals who reported that past residential and educational environments were mostly white would have higher adult health literacy and that individuals who reported that current residential, work, and social environments were mostly white would have higher adult health literacy. These findings can inform our understanding of how social determinants of health over the life course impact health literacy.

## Ethics Approval and Consent to Participate

All study participants provided oral and written consent and had the option to merge their survey responses with their medical records. The primary data collection was approved by the Human Research Protection Office at the Washington University School of Medicine, and secondary data analysis was approved by the New York University Institutional Review Board (IRB).

## Data Availability

The data supporting the findings of this study are from the Survey of Center for Outpatient Health Patients and can be obtained by making a request to the corresponding author through the following link: https://publichealth.nyu.edu/w/casjph/cohp. Access to the data requires IRB approval because the data contains information (e.g., medical records) that could compromise the privacy of study participants.

## Methods

### Setting

This study was conducted at the Barnes-Jewish Hospital Center for Outpatient Health (COH) primary care clinic in St. Louis, Missouri (Bather et al., 2024). In 1 year, this clinic served 16,907 unique patients, most of whom were Black (64%) or White (30%). Most of these patients were women (67%), between ages 35 and 64 years (59%), lived in St. Lou- is City or County (77%), and were covered by Medicare or Medicaid (80%).

### Data Collection

From July 2013 to April 2014, trained research assistants (RAs) approached 4,243 patients to complete a survey in the COH waiting room. To be eligible, participants had to be at least 18 years old, be a COH patient, and speak English. RAs then asked eligible participants to complete a self-administered written questionnaire that assessed demographic characteristics, followed by a verbally administered survey component that assessed health literacy and racial composition of past and current environments using measures described below. All participants provided verbal and written consent before completing the survey and received a small incentive for their participation. For initial Institutional Review Board approval of this study, the Human Research Protection Office at the Washington University School of Medicine approved this study. Secondary data analysis was approved by the New York University Institutional Review Board.

### Analytic Sample

**Figure 1.**
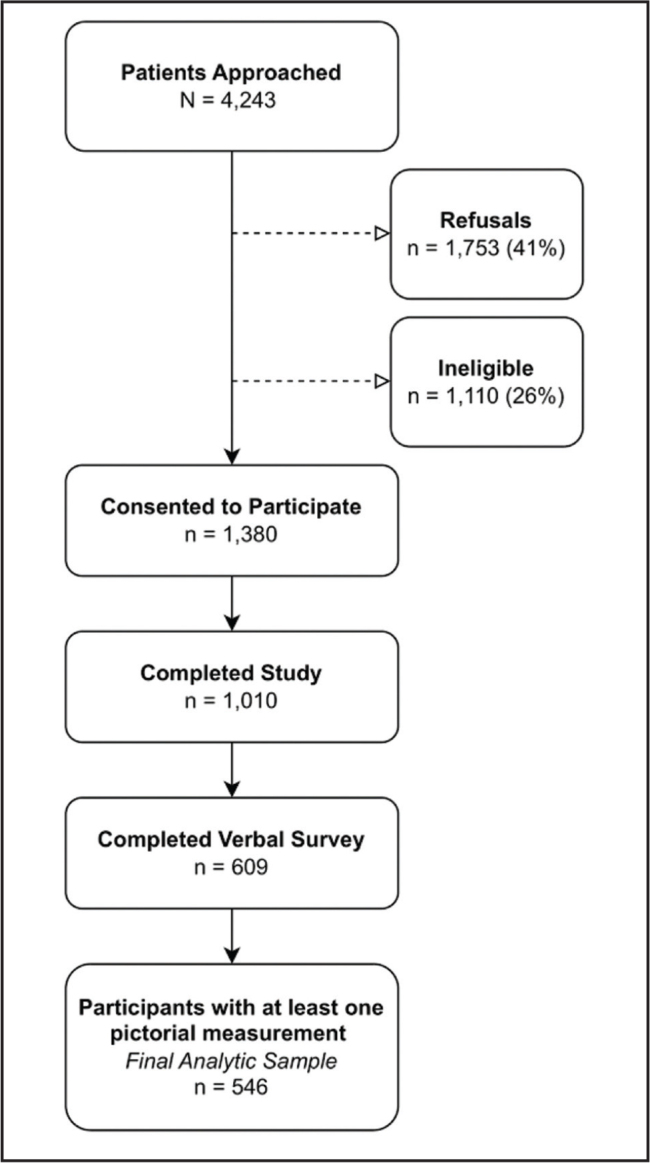
Study recruitment flowchart from a primary care clinic in St. Louis, Missouri.

**Figure 1** outlines the development of the final analytic sample. Of the 4,243 people approached, 26% (*n* = 1,110) were not eligible, and 41% (*n* = 1,753) refused to participate. Most people who refused did not provide a reason, while others reported a lack of interest, lack of time, or illness. Among eligible participants, 44% (*n* = 1,380) consented to participate. Of those 1,380 participants, 73% (*n* = 1,010) completed the written component of the questionnaire and 44% (*n* = 609) the verbal component. Of the 609 participants that completed written questionnaires, 546 (90%) had at least one pictorial measurement and were included in the final analytic sample. The primary reason for incomplete surveys was inadequate time between the start of the survey and when the clinic was ready to begin the patient evaluation.

### Measures

***Health literacy.*** We assessed health literacy with two validated measures. The first measure was the Rapid Estimate of Adult Literacy in Medicine-Revised (REALM-R) (Bass III et al., 2003; Davis et al., 1993). Based on standard scoring, we categorized participants who correctly pronounced more than six words as having adequate health literacy, and those pronouncing 0 to 6 words correctly as having limited health literacy (Agency for Healthcare Research and Quality, 2013). The second measure was the Newest Vital Sign (NVS) (Weiss et al., 2005). Using standard scoring, we categorized participants with scores 4 to 6 as having adequate health literacy, and those with scores 0 to 3 as having limited health literacy.

***Racial composition of social environments***. The primary predictor was a 10-item pictorial measure adapted from the work of Krysan and Farley (2002). As part of the verbal survey, RAs asked participants to identify the approximate racial composition of 10 social environments. The six past environments included block, neighborhood, junior high, junior high classroom, high school, and high school classroom. The four current environments included block, neighborhood, place of worship, and workplace. For each environment, participants selected 1 of 7 possible options: 100% Black, 70% Black, 50% Black, 30% Black, 10% Black, 100% White, and Not Applicable. To identify predominantly white social environments, we created indicator variables for the selection of the *30% Black*, *10% Black*, or *100% White* options. Participants' responses indicating *Not Applicable* or *Did not go to high school (or junior high school) in the US* were coded as missing. For past and current environments, we summed indicator variables to create final dichotomous measures of those with two or more vs. those with one or no predominantly white social environments.

***Demographic characteristics.*** We assessed several demographic characteristics as possible covariates correlated with health literacy. Age and neighborhood poverty (% under the poverty level) were measured continuously. Using the participant's self-reported zip code, we extracted neighborhood poverty rates from the 2012 American Community Survey. Race and ethnicity were categorized as White, Black (including multiracial), or other/unknown. Sex assigned at birth was categorized as male or female. Educational attainment was categorized as less than high school, high school/general education diploma, or some college education or more. Employment status was categorized as working or not in the workforce. Household income was categorized as <$20,000 or ≥$20,000. Location of residence was categorized as St. Louis City, St. Louis County, or other. Health insurance coverage was categorized as none, public, or private. Self-reported health status was dichotomized as *fair/poor* vs. *excellent/very good/good*.

### Statistical Analysis

We calculated descriptive statistics for all health literacy, racial composition, and demographic variables and scales. We used t-tests and chi-squared tests to examine bivariate associations with the past and current racial composition of social environments and demographic variables. Demographic characteristics with a bivariate association of *p* < .10 were included in final adjusted multilevel logistic regression models (Raudenbush & Bryk, 2002). These models accounted for the correlation between participants within the same zip code. We present odds ratios with their 95% confidence intervals. R was used for all analyses (R Core Team, 2023), with statistical significance assessed as *p* < .05.

## Results

### Participant Characteristics

As shown in **Table 1**, the average age of the study participants was 51 years (standard deviation = 12). Almost two-thirds (61%) identified as Black or multiracial, 32% as White, and 7% as another racial and ethnic category or unknown. The majority identified as female (66%), were not working (83%), and had a household income of <$20,000 (72%). The majority (54%) had a high school/general education diploma or less. On average, about 25% of the population in the participant's zip code lived under the poverty level. Most participants (55%) had public health insurance and rated their health status as *fair/poor *(61%). Proportions scored as having adequate health literacy were 56% based on the REALM-R and 38% based on the NVS.

**Table 1:**
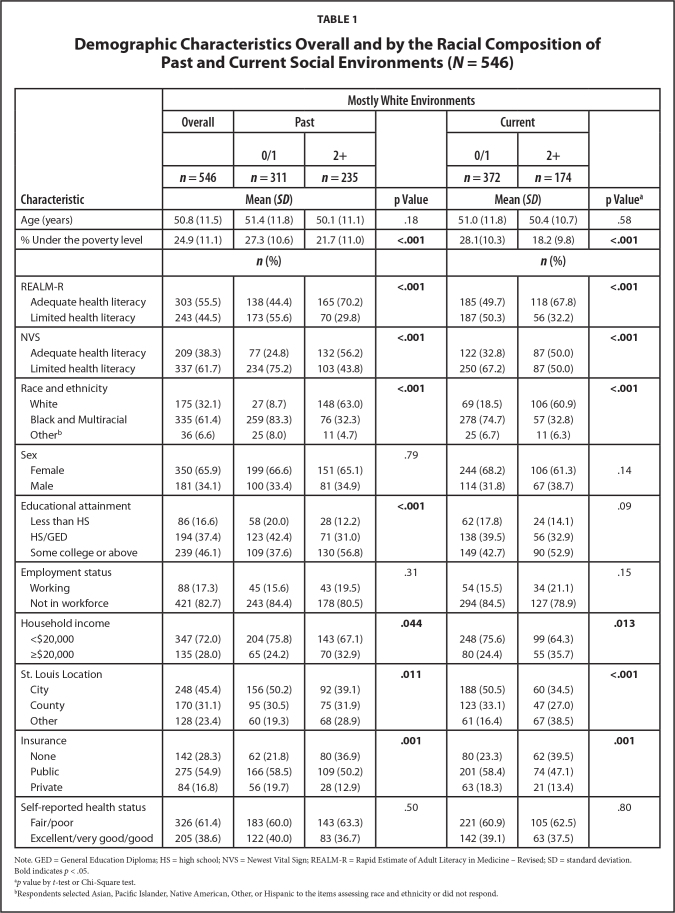
Demographic Characteristics Overall and by the Racial Composition of Past and Current Social Environments (*N* = 546)

**Characteristic**	**Mostly White Environments**						

	**Overall**	**Past**			**Current**		
	
		**0/1**	**2+**		**0/1**	**2+**	
	
	***n* = 546**	***n* = 311**	***n* = 235**		***n* = 372**	***n* = 174**	

	**Mean (*SD*)**			**p Value**	**Mean (*SD*)**		**p Value^a^**

Age (years)	50.8 (11.5)	51.4 (11.8)	50.1 (11.1)	.18	51.0 (11.8)	50.4 (10.7)	.58

% Under the poverty level	24.9 (11.1)	27.3 (10.6)	21.7 (11.0)	**<.001**	28.1(10.3)	18.2 (9.8)	**<.001**

	***n* (%)**				***n* (%)**		

REALM-R				**<.001**			**<.001**
Adequate health literacy	303 (55.5)	138 (44.4)	165 (70.2)		185 (49.7)	118 (67.8)	
Limited health literacy	243 (44.5)	173 (55.6)	70 (29.8)		187 (50.3)	56 (32.2)	

NVS				**<.001**			**<.001**
Adequate health literacy	209 (38.3)	77 (24.8)	132 (56.2)		122 (32.8)	87 (50.0)	
Limited health literacy	337 (61.7)	234 (75.2)	103 (43.8)		250 (67.2)	87 (50.0)	

Race and ethnicity				**<.001**			**<.001**
White	175 (32.1)	27 (8.7)	148 (63.0)		69 (18.5)	106 (60.9)	
Black and Multiracial	335 (61.4)	259 (83.3)	76 (32.3)		278 (74.7)	57 (32.8)	
Other^b^	36 (6.6)	25 (8.0)	11 (4.7)		25 (6.7)	11 (6.3)	

Sex				.79			
Female	350 (65.9)	199 (66.6)	151 (65.1)		244 (68.2)	106 (61.3)	.14
Male	181 (34.1)	100 (33.4)	81 (34.9)		114 (31.8)	67 (38.7)	

Educational attainment				**<.001**			.09
Less than HS	86 (16.6)	58 (20.0)	28 (12.2)		62 (17.8)	24 (14.1)	
HS/GED	194 (37.4)	123 (42.4)	71 (31.0)		138 (39.5)	56 (32.9)	
Some college or above	239 (46.1)	109 (37.6)	130 (56.8)		149 (42.7)	90 (52.9)	

Employment status				.31			.15
Working	88 (17.3)	45 (15.6)	43 (19.5)		54 (15.5)	34 (21.1)	
Not in workforce	421 (82.7)	243 (84.4)	178 (80.5)		294 (84.5)	127 (78.9)	

Household income				**.044**			**.013**
<$20,000	347 (72.0)	204 (75.8)	143 (67.1)		248 (75.6)	99 (64.3)	
≥$20,000	135 (28.0)	65 (24.2)	70 (32.9)		80 (24.4)	55 (35.7)	

St. Louis Location				**.011**			**<.001**
City	248 (45.4)	156 (50.2)	92 (39.1)		188 (50.5)	60 (34.5)	
County	170 (31.1)	95 (30.5)	75 (31.9)		123 (33.1)	47 (27.0)	
Other	128 (23.4)	60 (19.3)	68 (28.9)		61 (16.4)	67 (38.5)	

Insurance				**.001**			**.001**
None	142 (28.3)	62 (21.8)	80 (36.9)		80 (23.3)	62 (39.5)	
Public	275 (54.9)	166 (58.5)	109 (50.2)		201 (58.4)	74 (47.1)	
Private	84 (16.8)	56 (19.7)	28 (12.9)		63 (18.3)	21 (13.4)	

Self-reported health status				.50			.80
Fair/poor	326 (61.4)	183 (60.0)	143 (63.3)		221 (60.9)	105 (62.5)	
Excellent/very good/good	205 (38.6)	122 (40.0)	83 (36.7)		142 (39.1)	63 (37.5)	

Note. GED = General Education Diploma; HS = high school; NVS = Newest Vital Sign; REALM-R = Rapid Estimate of Adult Literacy in Medicine – Revised; SD = standard deviation.

Bold indicates *p* < .05.

a *p* value by *t*-test or Chi-Square test.

b Respondents selected Asian, Pacific Islander, Native American, Other, or Hispanic to the items assessing race and ethnicity or did not respond.

### Participant Characteristics by Racial Composition of Social Environments

We examined the bivariate relationships between demographic characteristics and the racial composition of past social environments (**Table 1**). Compared to participants with one or no past white social environments, those with multiple white environments had higher health literacy (REALM-R: 70% vs. 44%; NVS: 56% vs. 25%; both *p* < .001), higher educational attainment (57% vs. 38% with some college education, *p* < .001), higher household income (33% vs. 24% with ≥$20,000, *p* = .044), and were more likely to be White (63% vs. 9%, *p* < .001). Participants with multiple past white environments were also less likely to live in the city (39% vs. 50%, *p* = .011), less likely to have public health insurance (50% vs. 59%, *p* = .001), and less likely to live in areas with lower poverty rates (22% vs. 27%, *p* < 0.001). We did not observe significant differences in racial composition of past environments by age, sex, employment status, or self-reported health. Patterns of associations between these variables and the racial composition of current social environments were similar, except for educational attainment (**Table 1**). Participants with multiple current white environments did not differ significantly from those with one or none with regard to educational attainment (*p* = .09).

### Multivariable Predictors of Adequate Health Literacy

**Table 2** presents the results of the multivariable analysis of racial composition of social environments and REALM-R. We found that the racial composition of past social environments was significantly associated with health literacy as assessed by REALM-R. Participants with multiple past white environments (vs. 0 or 1) were more likely to have adequate health literacy (adjusted *OR* = 1.79; 95% CI: 1.04–3.07). We did not find a significant relationship between the racial composition of current social environments and health literacy (aO*R* = 1.07; 95% CI: 0.60–1.93). However, both models showed that race and ethnicity, educational attainment, and neighborhood poverty were significantly associated with adequate health literacy. Regarding race, Black and multiracial participants (past aO*R* = 0.45, 95% CI: 0.23–0.86; current aO*R* = 0.32, 95% CI: 0.18–0.56) were less likely than White participants to have adequate health literacy. Participants with some college or higher level of education (past aO*R* = 4.08, 95% CI: 2.11–7.87; current aO*R* = 4.45, 95% CI: 2.33–8.49) were more likely to have adequate literacy than those who did not have a high school diploma. Additionally, higher poverty rates was associated with limited health literacy (both aO*R* = 0.97, 95% CI: 0.94–0.99). Household income, St. Louis location, and insurance were not significantly associated with health literacy in either model.

**Table 2:**
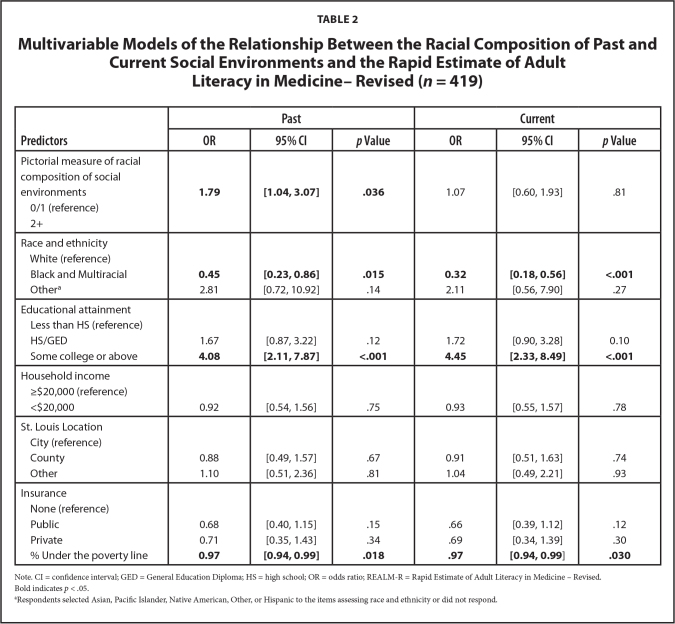
Multivariable Models of the Relationship Between the Racial Composition of Past and Current Social Environments and the Rapid Estimate of Adult Literacy in Medicine–Revised (*n* = 419)

**Predictors**	**Past**			**Current**		

	**OR**	**95% CI**	***p* Value**	**OR**	**95% CI**	***p* Value**

Pictorial measure of racial composition of social environments	**1.79**	**[1.04, 3.07]**	**.036**	1.07	[0.60, 1.93]	.81
0/1 (reference)						
2+						

Race and ethnicity						
White (reference) Black and Multiracial	**0.45**	**[0.23, 0.86]**	**.015**	**0.32**	**[0.18, 0.56]**	**<.001**
Other^a^	2.81	[0.72, 10.92]	.14	2.11	[0.56, 7.90]	.27

Educational attainment						
Less than HS (reference) HS/GED	1.67	[0.87, 3.22]	.12	1.72	[0.90, 3.28]	0.10
Some college or above	**4.08**	**[2.11, 7.87]**	**<.001**	**4.45**	**[2.33, 8.49]**	**<.001**

Household income						
≥$20,000 (reference)						
<$20,000	0.92	[0.54, 1.56]	.75	0.93	[0.55, 1.57]	.78

St. Louis Location						
City (reference)						
County	0.88	[0.49, 1.57]	.67	0.91	[0.51, 1.63]	.74
Other	1.10	[0.51, 2.36]	.81	1.04	[0.49, 2.21]	.93

Insurance						
None (reference)						
Public	0.68	[0.40, 1.15]	.15	.66	[0.39, 1.12]	.12
Private	0.71	[0.35, 1.43]	.34	.69	[0.34, 1.39]	.30
% Under the poverty line	**0.97**	**[0.94, 0.99]**	**.018**	**.97**	**[0.94, 0.99**]	**.030**

Note. CI = confidence interval; GED = General Education Diploma; HS = high school; OR = odds ratio; REALM-R = Rapid Estimate of Adult Literacy in Medicine – Revised.

Bold indicates *p* < .05.

a Respondents selected Asian, Pacific Islander, Native American, Other, or Hispanic to the items assessing race and ethnicity or did not respond.

**Table 3** presents the multivariable models with health literacy assessed by the NVS as the outcome. The model with past social environments as the predictor of interest showed that participants who had multiple white social environments were almost twice as likely (aO*R* = 1.94, 95% CI: 1.15–3.27) to have adequate health literacy than those who had one or no white social environments. The model with current social environments (aO*R* = 0.82, 95% CI: 0.47–1.44) did not show a significant association with health literacy, similar to the REALM-R model. Also similar to the REALM-R models were the significant predictors of race and ethnicity, educational attainment, and neighborhood poverty in both the past and current environments models. Black and multiracial participants were less likely than White participants to have adequate health literacy (past aO*R* = 0.48, 95% CI: 0.26–0.88; current aO*R* = 0.31, 95% CI: 0.18–0.53). Those with at least some college education were more likely than those without a high school diploma to have adequate health literacy (past aO*R* = 2.56, 95% CI: 1.31–4.99; current aO*R* = 2.84, 95% CI: 1.48–5.47), and higher poverty rates was associated with limited health literacy (past aO*R* = 0.97, 95% CI: 0.94–0.99; current aO*R* = 0.96, 95% CI: 0.93–0.99). In the current environments model, we also found that individuals with public health insurance (aO*R* = 0.60, 95% CI: 0.37–0.98) were less likely than those without health insurance to have adequate health literacy. We did not find significant associations between health literacy with health insurance, household income or St. Louis location.

**Table 3:**
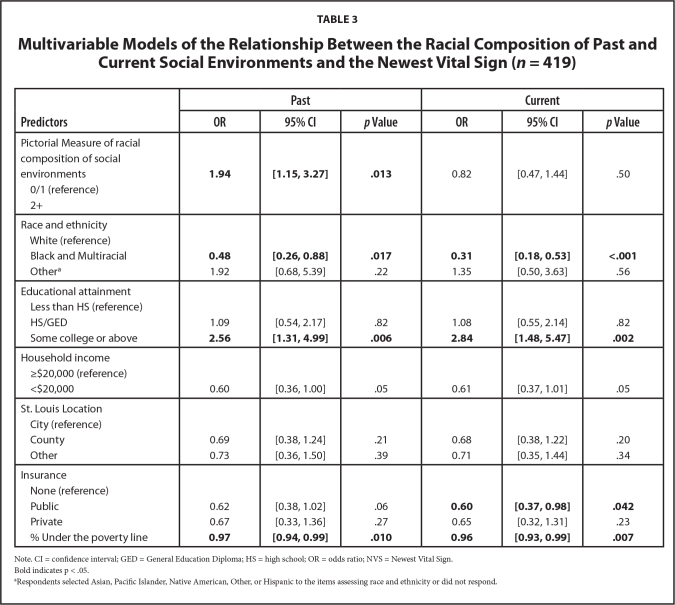
Multivariable Models of the Relationship Between the Racial Composition of Past and Current Social Environments and the Newest Vital Sign (*n* = 419)

**Predictors**	**Past**			**Current**		

	**OR**	**95% CI**	***p* Value**	**OR**	**95% CI**	***p* Value**

Pictorial Measure of racial composition of social environments	**1.94**	**[1.15, 3.27]**	**.013**	0.82	[0.47, 1.44]	.50
0/1 (reference)						
2+						

Race and ethnicity						
White (reference)						
Black and Multiracial	**0.48**	**[0.26, 0.88]**	**.017**	**0.31**	**[0.18, 0.53]**	**<.001**
Other^a^	1.92	[0.68, 5.39]	.22	1.35	[0.50, 3.63]	.56

Educational attainment						
Less than HS (reference)						
HS/GED	1.09	[0.54, 2.17]	.82	1.08	[0.55, 2.14]	.82
Some college or above	**2.56**	**[1.31, 4.99]**	**.006**	**2.84**	**[1.48, 5.47]**	**.002**

Household income						
≥$20,000 (reference)						
<$20,000	0.60	[0.36, 1.00]	.05	0.61	[0.37, 1.01]	.05

St. Louis Location						
City (reference)						
County	0.69	[0.38, 1.24]	.21	0.68	[0.38, 1.22]	.20
Other	0.73	[0.36, 1.50]	.39	0.71	[0.35, 1.44]	.34

Insurance						
None (reference)						
Public	0.62	[0.38, 1.02]	.06	**0.60**	**[0.37, 0.98]**	**.042**
Private	0.67	[0.33, 1.36]	.27	0.65	[0.32, 1.31]	.23
% Under the poverty line	**0.97**	**[0.94, 0.99]**	**.010**	**0.96**	**[0.93, 0.99]**	**.007**

Note. CI = confidence interval; GED = General Education Diploma; HS = high school; OR = odds ratio; NVS = Newest Vital Sign.

Bold indicates p < .05.

a Respondents selected Asian, Pacific Islander, Native American, Other, or Hispanic to the items assessing race and ethnicity or did not respond.

## Discussion

Our findings highlight the impact of the racial composition of past social environments on adult health literacy, suggesting that resources available to children in their educational and residential social settings have lifelong effects. Participants who had multiple white social environments in past residential and educational contexts were significantly more likely to have adequate health literacy as adults than those who had not, whether health literacy was assessed with the REALM-R or NVS. In contrast, participants reporting having multiple white social environments in current contexts were not more likely to have adequate health literacy than other adults. These prior findings combined with our results, therefore, suggest the importance of longitudinal models of how social determinants of health over the life course impact health literacy.

Mechanisms by which the racial composition of past social environment might impact an individual's health literacy as an adult have been suggested by prior work. While this has not yet been studied in the context of health literacy, childhood environments have been shown to impact adult health behaviors and health outcomes (Caughy et al., 2003; Chen et al., 2022; Chi et al., 2017; Lynch & Meier, 2020; Root & Humphrey, 2014). Structural-level determinants have been shown to have persistent and intergenerational effects. In countries such as the United States and Germany, there are low levels of social mobility for education, meaning that the level of educational attainment is an intergenerational phenomenon (Lunze & Paasche-Orlow, 2014). Other research has extensively documented the intergenerational effects of systemic racism and enforced segregation in residential and educational contexts on numerous outcomes, including health and education (Bailey et al., 2017; Bonilla-Silva, 2021; Kramer & Hogue, 2009; Lipsitz, 2011; Massey & Denton, 1993; Tatum, 2017; Washington, 2006; Williams, 2012; Williams & Collins, 2001; Williams et al., 2019). The findings of the present study suggest an important impact of these contexts on adult health literacy, which may mediate the downstream effects on health outcomes and health disparities.

Our findings are consistent with prior health literacy literature showing correlations between individual characteristics and health literacy, independent of the effects of racial composition of past or current social environments. As has been observed in previous studies (Kutner et al., 2006; Nutbeam & Lloyd, 2021; Paasche-Orlow et al., 2005), in our study, Black participants were less likely to have adequate health literacy than White participants, and those with higher educational attainment were more likely to have adequate health literacy than those with lower educational attainment. The associations between neighborhood-level characteristics and health literacy have been less investigated than those between individual-level characteristics and health literacy. Our study, therefore, adds to the existing literature with the observation that neighborhood poverty as an adult was associated with health literacy and that this effect was independent of the effects of racial composition of social environments or individual-level demographic characteristics. Our findings highlight the importance of investigating multilevel variables upstream of health literacy, including both system-level and individual-level factors.

Although the associations between racial composition of social environments and health literacy were similar for both measures of health literacy, we did observe some differences. Fewer adults were categorized as having adequate health literacy with the NVS compared to the REALM-R, as we have found previously (Carpenter et al., 2014). Based on our prior work, this may be due, at least in part, to the numeracy components of the NVS (Carpenter et al., 2014; Griffey et al., 2014). We also found similar but not identical correlates of health literacy in models in which health literacy was assessed with the NVS compared to the REALM-R. These findings reinforce the importance of examining predictors of different dimensions of health literacy (Davis et al., 1993; Mancuso, 2009; Weiss et al., 2005). In addition, it will be important in future studies to examine the associations between social determinants of health and measures that capture other dimensions, such as critical health literacy (i.e., individuals' understanding of the social determinants of health combined with the skills to take action at both the individual and community level (Chinn, 2011; Mogford et al., 2011).

## Study Limitations

Our findings should be considered in light of the study's limitations. The study was conducted in a single geographic area, and nationally representative data are needed to investigate these associations further. The patient population of the clinic was mostly Black or White, and it will be important to expand these findings to other racial and ethnic groups in future studies. In addition, our study was cross-sectional; while the data indicate the importance of longitudinal designs to examine the impact of social environments on health literacy over the life course, we were not able to do so directly. The survey was administered in English, and it will be important to also investigate the impact of social determinants of health on health literacy among individuals who speak other languages. Finally, self-reported racial composition may differ from objective racial composition of past and current environments, and measurement of this construct is important to examine further.

## Conclusion

Despite these limitations, our findings add to the existing literature on determinants of health literacy by showing an association between the racial composition of past educational and residential social environments and adult health literacy. The results highlight the importance of examining social determinants over the life course for their impact on health literacy, as well as downstream health outcomes and health disparities. The findings also indicate that policies ensuring equitable access to educational resources in school and community contexts are critical to improving equitable health literacy, and thereby health outcomes, for adults. Addressing disparities in health outcomes will likely require system-level policy solutions, an important area for future health literacy research.
